# Deciphering the Bioactive Landscape of *Satureja nepeta* Essential Oil: A Synergistic Exploration of Its Antimicrobial, Antiproliferative Potentials

**DOI:** 10.3390/life16071115

**Published:** 2026-07-03

**Authors:** Houssam Assioui, Kaouthar Elbirgui, Othmane El Faqer, Wafaa Taha, Fatima Zahra Kadiri, Mariame Elmessal, Faiza Bennis, Jean-François Landrier, Fatima Chegdani

**Affiliations:** 1 Laboratory of Integrative Biology, Faculty of Sciences Aïn Chock, Hassan II University of Casablanca, Casablanca 20100, Morocco; houssam.assioui@etu.univ-amu.fr (H.A.);; 2 Aix-Marseille Université, C2VN, INSERM, INRAE, 13000 Marseille, France; 3 Laboratory of Chemistry and Biochemistry, Nutrition, Environment and Health, Faculty of Medicine and Pharmacy of Casablanca, Casablanca 20250, Morocco; 4 Mohammed VI Polytechnic University, Benguerir 43150, Morocco

**Keywords:** *Satureja nepeta*, GC-MS chemical profiling, antioxidant activity, antibacterial properties, molecular docking, antiproliferative effect

## Abstract

*Satureja nepeta* essential oil (EO) is gaining prominence for its multifaceted pharmacological and biotechnological potential. This study aimed to characterize its volatile profile and evaluate its functional capacity as an antioxidant, antibacterial, and antiproliferative agent. Gas Chromatography Mass Spectrometry (GC–MS) profiling was conducted to identify the volatile constituents of the EO. Antioxidant activity was assessed using DPPH, ABTS, TAC, and FRAP assays. Antibacterial activity was evaluated against Gram-positive and Gram-negative pathogens using disk diffusion and MIC determination. In silico molecular docking against bacterial DNA gyrase B was performed to explore potential mechanisms of action. Antiproliferative activity was assessed on the P3X63Ag8.653 myeloma cell line. Chemical profiling identified nine major constituents, dominated by pulegone (68.63%), menthol (6.64%), and cis-pulegol (2.04%). The EO demonstrated significant free radical-scavenging activity, particularly in the TAC assay (EC_50_ = 3.747 ± 0.577 µg/mL). Antimicrobial evaluations revealed robust inhibitory effects, with *Pseudomonas aeruginosa* and *Salmonella enterica* exhibiting the highest susceptibility. In silico modeling corroborated these findings, identifying menthol as the lead ligand (ΔG = −6.09 kcal/mol), suggesting a synergistic mechanism of action. Notably, the EO displayed potent antiproliferative activity (LC_50_ = 14.060 ± 1.364 µg/mL), falling well within the high-cytotoxicity threshold. Collectively, these findings underscore the pharmacological significance of *S. nepeta* EO as a potent reservoir of bioactive monoterpenes with antioxidant, antimicrobial, and anticancer properties, meriting further in vivo validation and mechanistic exploration toward its development as a therapeutic or nutraceutical candidate.

## 1. Introduction

The use of plant-derived essential oils (EOs) [1] has gained widespread recognition for its ability to support physical, emotional, and mental well-being. Its effectiveness stems from the rich diversity of natural compounds present in EOs [2]. EOs obtained from a wide range of aromatic plants play a central role in traditional pharmacopoeias, particularly in Mediterranean and tropical regions. These products are volatile, generally transparent liquids; sometimes slightly colored; lipophilic and soluble in organic solvents; and with a density usually lower than that of water [3].

Among aromatic plants, *Satureja nepeta* (syn. *Calamintha nepeta*), a perennial aromatic species belonging to the Lamiaceae (Labiatae) family, stands out for the richness and diversity of its EOs [4]. Native to the Mediterranean region, this species is highly appreciated for its broad spectrum of biological activities and its longstanding use in traditional medicine. In the Al Haouz region of Morocco, it is particularly employed for the management of respiratory infections and intestinal inflammatory disorders. Several studies have attributed the biological properties of *S. nepeta* to its richness in monoterpenes and sesquiterpenes, although the relative abundance of these compounds varies considerably among populations [5]. Previous investigations have revealed considerable variability in the chemical composition of *S. nepeta* EO, a feature commonly observed among members of the Lamiaceae family. El Brahimi et al. identified 42 compounds representing 98.96% of the total oil, with eucalyptol (23.10%), pulegone (12.44%), rotundifolone (9.68%), and spathulenol (6.52%) as the major constituents [6]. Similarly, Radi et al. reported pulegone (87.04%) as the dominant compound in *S. nepeta* EO collected from the Meknes region of Morocco [7]. In contrast, Hayani et al. identified 1,8-cineole (34.34%) and cis-pinocamphone (11.87%) as the principal constituents of EOs from the same region [8]. Studies conducted in Algeria also reported distinct chemical profiles, with trans-menthone and pulegone predominating in samples from Bejaia [9], whereas pulegone and isomenthone were identified as the major constituents in western Algerian populations [10]. Likewise, Lebanese *S. nepeta* EO was characterized by the predominance of isomenthone, menthone, pulegone, α-bisabolol, hexadecanoic acid, and β-eudesmol [11]. Such qualitative and quantitative variations are generally attributed to differences in geographical origin, climatic conditions, genetic polymorphism, phenological stage, drying procedures, and extraction methods [11].

Given this marked chemical variability and its potential impact on biological properties, further investigations of *S. nepeta* populations from different geographical regions remain of considerable interest. Therefore, this study aims to chemically characterize the EO of *S. nepeta* collected from the Al Haouz region of Morocco by identifying its major bioactive compounds. In addition, its antioxidant, antibacterial, and antiprolifertive activities were evaluated to provide further insights into its biological potential and to support the traditional uses of this species while exploring its possible applications in pharmaceutical and healthcare fields.

## 2. Materials and Methods

### 2.1. Plant Material

The collection of *S. nepeta* was realized in May 2023 from the El-Haouz region (latitude 31°13′55.6″ N/longitude 7°49′56.0″ W). The plant identification was carried out at The Research Institute in Rabat, Morocco. The specimen voucher number, RAB 114765, has been deposited in the herbarium in the scientific Institute and in the Laboratory of Integrative Biology, Department of Biology, at the Faculty of Science Ain-chock, University Hassan II, of Casablanca, Morocco.

### 2.2. Essential Oil Preparation

The plant material was air-dried for 7 days, minimizing exposure to light and humidity to preserve its phytochemical integrity. It was then subjected to cryogenic grinding using liquid nitrogen to prevent the degradation of volatile compounds. EO extraction was carried out via hydro-distillation for 4 h from 100 g of the areal part using a Clevenger apparatus, following the protocols outlined in the European Pharmacopoeia [12]. The EO yield was calculated on a weight-to-weight basis (*w*/*w*) relative to the dry weight of the plant material. The obtained EO was stored at −20 °C in the dark to maintain its chemical stability until analysis. The extraction was performed in triplicate to ensure the reproducibility of the results.

### 2.3. GC-MS Profiling

The EO was analyzed at the analysis center of the Faculty of Sciences Ain Chock (Casablanca, Morocco), using a Shimadzu GC-2010 gas chromatograph (Shimadzu, Istanbul, Turkey) coupled with a QP2010 Plus mass spectrometer (software version 2.50 SU1) according to El Faqer et al. [13]. The system was equipped with a BP-5 capillary column (30 m × 0.25 mm × 0.25 μm; SGE, Ltd.). The oven temperature was programmed per GC protocols, with transfer line and ion source temperatures set at 300 °C and 200 °C, respectively. Helium was used as the carrier gas at 36.5 cm/s, with a split ratio of 1:40, ionization energy of 70 eV, and a scan range of 40–400 amu. Compounds were identified by comparing Kovats indexes, a standardized measure that allows for precise compound identification in GC-MS regardless of experimental conditions, using C9-C20 n-alkanes and confirming mass spectra against the Shimadzu library, NIST05 database, and an in-house reference library based on standards, synthetic components, and literature data [14].

### 2.4. Assessment of Antioxidant Properties of S. nepeta

#### 2.4.1. DPPH Radical Scavenging Activity

The DPPH (diphenylpicrylhydrazyl) test is widely used to analyze antioxidant activity. DPPH produces stable free radicals due to the delocalization of free electrons within the molecule, resulting in a deep violet color that absorbs around 517 nm. When an antioxidant reduces the DPPH radicals, the solution turns yellow, decreasing absorbance. The antioxidant potential of a sample is often expressed by the IC_50_ value, which is the concentration needed to reduce the initial DPPH concentration by 50% [15].

The 2,2-diphenyl-1-picrylhydrazyl (DPPH) assay was carried out according to the protocol described by Bendiar et al. [16], with some modifications. Briefly, 50 µL of EO with different concentrations was added to 1.95 mL of 60 µM DPPH ethanol solution. The test tubes were shaken vigorously and incubated in the dark for 30 min at room temperature. The absorbance was measured at 517 nm for all the test tubes. Ascorbic acid was used as the reference standard. The scavenging activity was calculated using the following equation for DPPH scavenging activity:

$$\%\;Inhibition=\frac{A0-At}{A0}\times 100$$

where A0 is the absorbance of the control, and At is the absorbance of each sample.

The results of antiradical DPPH activity of the different types of extracts were expressed as IC_50_ in (mg/mL).

#### 2.4.2. ABTS Radical Scavenging Assay

The basis of this assay lies in the reduction of the ABTS^+^ radical cation to ABTS. Mixing ABTS with potassium persulfate generates the blue-green ABTS^+^ radical cation. When an antioxidant sample is introduced, the ABTS^+^ radical cation is reduced back to ABTS [17].

ABTS radical cation assay was carried out using the protocol of Elkoraichi et al. [18]. A total of 7.4 mM of ABTS was mixed with 2.45 mM of potassium persulphate (K_2_S_2_O_8_) solution and then left in the dark for 18 h to facilitate the production of free radicals (ABTS^+^). A milliliter of this resulting solution was then diluted using 50 mL of ethanol, which created a working solution with an absorbance level of 0.700 at 734 nm. EO and ascorbic acid at different concentrations were added to 1 mL of ABTS^+^ and left to interact with it in a darkened environment for 10 min. The absorbance was determined at 734 nm against ethanol as a blank:

$$\%\;ABTS=\frac{Abs\;control-Abs\;sample}{Abs\;control}\times 100$$

where Abs control represents the absorbance of ABTS^+^, while Abs sample signifies the absorbance of the samples.

The results of antiradical ABTS activity were expressed as IC_50_ in (mg/mL).

#### 2.4.3. Phosphomolybdenum Assay

This method is based on the reduction of molybdenum (Mo VI) present as molybdate ions (MoO_4_^2−^) to molybdenum (Mo V) (MoO^2+^) in the presence of the sample, resulting in the formation of a green phosphate/Mo(V) complex under acidic pH conditions [17,19]. To prepare the reagent solution, 4 mM ammonium molybdate (NH_4_)6Mo_7_O_24_, 28 mM of sodium phosphate (Na_3_PO_4_), and 0.6 M of sulphuric acid (H_2_SO_4_) were mixed. A total of 100 µL of EO was added to 1 mL of reagent solution and incubated at 95 °C for 90 min. Ascorbic acid was used as the reference standard. The samples were cooled at room temperature, and the absorbance was measured at 765 nm against a blank. The results were expressed as EC_50_ in (mg/mL).

#### 2.4.4. Reducing Power Determination

This assay is designed to assess the reducing potential of antioxidants. It involves converting ferric ions (Fe^3+^) within the potassium ferricyanide complex (K_3_[Fe(CN)_6_]) to ferrous ions (Fe^2+^), resulting in the formation of Prussian blue, which exhibits maximal absorption at λ = 700 nm when reducers are present in the tested extracts. Consequently, an elevated formation of this complex indicates a heightened reducing capacity of the sample being tested, thus determining its antioxidant potential.

The reducing power of the extracts was determined by the method of Samiry et al. [19]. Briefly, 1 mL of EO at different concentrations was mixed with 2.5 mL of phosphate buffer (0.2 M, pH 6.6) and 2.5 mL of 1% of K_3_[Fe(CN)_6_] and was incubated at 50 °C for 20 min. A total of 2.5 mL of trichloroacetic acid (10%) was added and centrifuged for 10 min, and 2.5 mL of supernatant was mixed with 2.5 mL of distilled water and 0.5 mL of FeCl_3_ (0.1%). Ascorbic acid was used as the reference standard. The absorbance of all samples was measured at 700 nm. The results were expressed as EC_50_ in (mg/mL).

### 2.5. Antibacterial Activity

#### 2.5.1. Preparation of Bacterial Strains

Seven bacterial strains were used: *Escherichia coli* (*E. Coli*) ATCC 8739, *Staphylococcus aureus* (*S. aureus*) ATCC 6538, *Staphylococcus epidermis* (*S. epidermis*) ATCC 12228, *Staphylococcus enterica* (*S. enterica*) ATCC 13076, *Micrococcus luteus* (*M. luteus*) ATCC 9341, *Pseudomonas aeruginosa* (*P. aeruginosa*) ATCC 27853, and *Bacillus subtilis* (*B. subtilis*) ATCC 6633. All the bacterial strains were cultured on Mueller Hinton (MH) overnight at 37 °C.

#### 2.5.2. Aromatogram

To evaluate the antibacterial activity of the EO, the disc diffusion method was used as described by Kadiri et al. [20]. The plates were mass-seeded with 108 CFU/mL and left to dry for 5 min. Sterile cellulose discs (6 mm diameter) impregnated with 10 µL of pure EO were placed on the agar surface. The plates were first incubated at room temperature for 30 min, followed by incubation at 37 °C for 24 h. The diameter of the inhibition zone (DIZ) was measured in millimeters. The activity index (AI) was calculated with the following formula:

$$AI=\frac{Diameter\;of\;the\;inhibition\;zone\;of\;sample}{Diameter\;of\;the\;inhibition\;zone\;of\;antibiotics}$$

#### 2.5.3. Minimal Inhibitory Concentration

To determine minimal inhibitory concentration (MIC) for the EO, a micro-dilution broth test was carried out according to Boughroud et al. [21] using sterile 96-well microplates. The EO was not subjected to heat sterilization, autoclaving, or membrane filtration prior to testing, as essential oils are volatile and chemically complex hydrophobic mixtures whose composition may be altered by such treatments, potentially leading to the loss or retention of active constituents. Instead, all manipulations were conducted under aseptic conditions using sterile materials and culture media. In each well, 50 μL of Mueller–Hinton (MH) broth was dispensed. A two-fold serial dilution was then performed using 25 μL of EO diluted in 10% dimethyl sulfoxide (DMSO), followed by the addition of 50 μL of the bacterial suspension. To ensure the reliability of the assay, several controls were included: MH broth with bacterial inoculum, MH broth containing bacterial suspension and solvent, and uninoculated MH broth to verify the absence of contamination. After incubation at 37 °C for 24 h, 30 μL of 0.01% (*m*/*v*) resazurin solution, previously filtered through a 0.45 μm membrane, was added to each well, followed by an additional 3 h incubation period. Bacterial growth was assessed visually based on the color change of resazurin from blue-purple to red or colorless. The MIC was defined as the lowest EO concentration that completely inhibited bacterial growth, as indicated by the absence of color change.

### 2.6. Molecular Docking Analysis

AutoDock Tools (ADT; version 1.5.7) was utilized to gain a deeper understanding of the interactions between the identified compounds in *S. nepeta* EO and the crystal structures of bacterial target proteins. The chemical structures of these compounds were sourced from the PubChem database in SDF format and subsequently converted into PDBQT format using the Open Babel graphical interface. Meanwhile, the crystal structures of the bacterial target proteins were retrieved from the Protein Data Bank (https://www.rcsb.org/) in PDB format, as detailed in Table 1. Protein preparation was carried out using ADT software following a multi-step process. Initially, non-protein atoms and water molecules were removed from the PDB files to correct structural imperfections, such as missing atoms and residues, which also contributed to energy minimization. The next step involved the addition of polar hydrogen atoms to the macromolecules and the incorporation of Kollman charges to ensure accurate calculation of partial atomic charges in the PDBQT format. Gasteiger charges were assigned to the chemical structures of the ligands using ADT. For docking simulations, a cubic grid box (60 × 60 × 60 Å) with a spacing of 0.375 Å was set up, defining the active site of each target protein. The coordinates of these active sites are presented in Table 1. Molecular docking studies were performed using ADT version 1.5.7. The ligands, prepared in PDBQT format, were docked into the binding sites of the target proteins under ADT’s default parameters. The docking results were assessed based on binding energy (ΔGb) in kcal/mol and inhibition constant (Ki) in µM for each compound. To further explore the molecular interactions within the ligand–receptor complexes, Biovia Discovery Studio Visualizer 2021 was employed for visualization and analysis.

### 2.7. Antiproliferative Activity

#### 2.7.1. Cell Lines and Cell Culture

The murine multiple myeloma cell line P3X63Ag8.653 was obtained from the Laboratory of Genetic and Cellular Engineering, Faculty of Medicine and Pharmacy, Casablanca, Morocco. Cells were maintained in RPMI 1640 medium (Sigma-Aldrich, St. Louis, MO, USA), a standard medium designed for mammalian cell culture, supplemented with 10% fetal calf serum (FCS; Gibco BRL), providing essential growth factors and nutrients. Additionally, the medium was supplemented with 2 mM L-glutamine, a crucial amino acid for cellular metabolism, and 100 U/mL penicillin and 100 µg/mL streptomycin (Sigma-Aldrich) to prevent bacterial contamination. Cultures were incubated at 37 °C in a humidified atmosphere containing 5% CO_2_, simulating physiological conditions and maintaining optimal pH for cell growth. Cell viability was routinely assessed using the trypan blue dye exclusion assay, which distinguishes live cells from dead cells.

#### 2.7.2. MTT Assay

For treatment experiments, P3X63Ag8.653 cells were seeded and allowed to proliferate for 24 h, ensuring stable cell growth before exposure to the test compound. Subsequently, cells were treated with varying concentrations of *S. nepeta* EO for a duration of 48 h. In addition, cells were treated with melphalan, a known cytotoxic agent, as a positive control to confirm the sensitivity of the cells to a standard chemotherapeutic drug and validate the assay’s ability to detect cell death. Negative control cells were treated with an equivalent volume of vehicle (culture medium) to account for any potential effects of the medium itself.

Cell viability was determined using the 3-(4,5-dimethylthiazol-2-yl)-2,5-diphenyltetrazolium bromide (MTT) assay, a colorimetric method that measures mitochondrial activity, which is indicative of cell viability. Briefly, after treatment, cells were incubated with MTT solution, which is reduced by metabolically active cells to form insoluble formazan crystals. The resulting formazan crystals were then dissolved in dimethyl sulfoxide (DMSO), and the absorbance, which is proportional to the number of viable cells, was measured at 492 nm using a spectrophotometer.

#### 2.7.3. Statistical Analysis

The analyses were conducted using GraphPad Prism version 9. Data are expressed as means ± standard deviations (n = 9). Student’s *t*-test was employed to compare the parameters, with a significance threshold set at *p* < 0.05.

## 3. Results

### 3.1. Phytochemical Characterization of S. nepeta EO (GC-MS Analysis)

**Figure 1:**
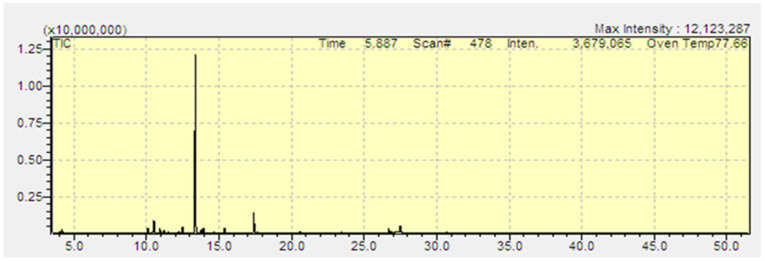
Gas chromatography–mass spectrometry (GC-MS) total ion chromatogram (TIC) of *S. nepeta* essential oil. The analysis was performed in full-scan mode, and each peak corresponds to a volatile compound separated by its retention time (min). The maximum signal intensity reached 1.21 × 10^7^ units.

In this study, we extracted and analyzed the chemical composition of *S. nepeta* EO using GC-MS. The results are summarized in Table 2 and Figure 1. The average oil yield was 0.076% (*w*/*w*). The GC-MS analysis identified a total of nine compounds, with pulegone as the predominant component (68.63%), followed by menthol (6.64%), cis-pulegol (2.04%), spathulenol (1.71%), piperitone (1.69%), isopulegone (1.58%), caryophyllene oxide (0.63%), neoisomenthol (0.57%), and isopiperitone (0.49%).

### 3.2. Antioxidant Activity of S. nepeta EO

During this study, we evaluated the antioxidant potential of *S. nepeta* EO using four distinct assays, and the results are shown in Table 3. *S. nepeta* EO effectively scavenged DPPH and ABTS^+^ radicals, and reduced the ferric/ferricyanide complex to the ferrous form and phosphate–molybdenum (VI) to phosphate–molybdenum (V) in its pure form with an immediate effect). Upon dilution, the EO retained moderate antioxidant activity, displaying IC_50_ and EC_50_ values exceeding 10 mg mL^−1^ in all assays except for TAC, where it achieved a markedly lower EC_50_ of 3.747 ± 0.577 µg mL^−1^ (Table 3). Relative to ascorbic acid (AA), the EO was markedly less active in DPPH and ABTS, more active in TAC, and essentially non-reducing in FRAP within the tested window. In DPPH, the EO showed an IC_50_ ≥ 10 mg/mL (≥10,000 µg/mL), whereas AA was 3.409 ± 0.196 µg/mL, indicating the EO is ≥2.9 × 10^3^-fold less potent. In ABTS, the EO IC_50_ was ≥10 mg/mL (≥10,000 µg/mL) versus 3.679 ± 0.763 µg/mL for AA (≥2.7 × 10^3^-fold less potent). By contrast, in TAC, the EO exhibited an EC_50_ of 3.747 ± 0.577 µg/mL compared with 7.42 ± 0.137 µg/mL for AA, with the EO being 2.0-fold more potent (lower EC_50_). In FRAP, the EO did not reach 50% effect (EC_50_ ≥ 10,000 µg/mL), while AA was 8.127 ± 0.467 µg/mL, a ≥1.23 × 10^3^-fold difference.

### 3.3. Antibacterial Activity of S. nepeta EO

#### 3.3.1. Aromatogram

The antibacterial activity of *S. nepeta* EO was evaluated against seven bacterial strains using the agar diffusion method, and the results are presented in Table 4. The EO exhibited antibacterial activity against all tested strains, with *P. aeruginosa* being the most sensitive, showing a diameter of inhibition zone (DIZ) of 27 ± 2.64 mm and an activity index (AI) of 0.87. *S. enterica* displayed a DIZ of 21.16 ± 0.28 mm, with an AI of 0.84, followed by *S. aureus* (DIZ = 21 ± 0 mm, AI = 0.77) and *S. epidermidis* (DIZ = 20 ± 0 mm, AI = 0.76). The EO exhibited a DIZ of 17 ± 0 mm and an AI of 0.77 against *M. luteus. B. subtilis* displayed moderate sensitivity, with a DIZ of 15.1 ± 0.17 mm and an AI of 0.83. *E. coli* was the most resistant strain, with a DIZ of 14 ± 0 mm and an AI of 0.77. Overall, the EO displayed broad-spectrum antibacterial activity, with AI values ranging between 0.76 and 0.87.

#### 3.3.2. Minimal Inhibitory Concentration (MIC)

The antibacterial activity of *S. nepeta* EO was further quantified through determination of the MIC against the tested bacterial strains (Table 4). The EO exhibited variable inhibitory potency, with MIC values ranging from 2.375 µg mL^−1^ to 104 µg mL^−1^. The strongest inhibition was recorded for *P. aeruginosa* (MIC = 2.375 µg mL^−1^), followed by *S. enterica* (3.25 µg mL^−1^), *S. aureus* (13.104 µg mL^−1^), and *S. epidermidis* (13.10 µg mL^−1^). Moderate susceptibility was observed for *M. luteus* (26 µg mL^−1^), while *E. coli* exhibited the lowest sensitivity (104 µg mL^−1^). These data confirm a strain-dependent antibacterial effect of *S. nepeta* EO, consistent with its broad-spectrum activity profile.

#### 3.3.3. Molecular Docking

The molecular docking analysis suggested moderate interactions between the tested monoterpenes and the DNA gyrase B active site of both Gram-positive (G^+^) and Gram-negative (G^−^) bacteria (Table 5). Among the evaluated compounds, menthol showed the most favorable predicted affinity toward the G^+^ gyrase B, with a binding energy of −6.09 kcal/mol and an estimated Ki of 34.41 µM. This docking pose was associated with a hydrogen-bond interaction involving ASN A:49. Pulegone and cis-pulegol exhibited slightly lower predicted affinities, with binding energies of −5.62 and −5.11 kcal/mol, respectively. Cis-pulegol was predicted to interact through hydrogen bonds with GLU A:53 and ASN A:56, whereas no hydrogen-bond interaction was observed for pulegone.

For the G^−^ gyrase B, the three compounds displayed similar predicted binding energies, ranging from −5.28 to −5.35 kcal/mol. Menthol and pulegone yielded the most favorable values (−5.34 and −5.35 kcal/mol, respectively), while cis-pulegol showed a slightly weaker predicted affinity (−5.28 kcal/mol). A hydrogen-bond interaction was predicted only for menthol, involving ASN A:46. The estimated Ki values ranged from 119.88 to 135.89 µM, suggesting generally weaker predicted interactions with the G^−^ enzyme than with the G^+^ counterpart.

Overall, the docking results may indicate a somewhat greater affinity of menthol for gyrase B, particularly in the G^+^ model. However, these observations should be interpreted as computational predictions and would require experimental validation to confirm any potential inhibitory effect on enzyme activity.

#### 3.3.4. Antiproliferative Activity of S. nepeta EO

**Figure 2:**
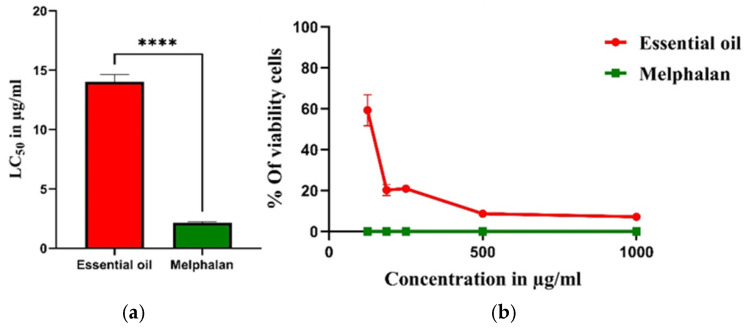
Evaluation of antiproliferative activity of *S. nepeta* EO on P3 cancer cell lines using MTT assay. (**a**) Percentage of viability cells; (**b**) determination of the 50% lethal concentration (LC_50_) for the essential oil compared to melphalan (control). ****, *p* < 0.0001 LC_50_ of *S. nepeta* EO vs. LC_50_ melphalan, determined by the *t*-test.

In this study, we evaluated the potential antiproliferative effect of *S. nepeta* EO on myeloma cell lines (P3) using the MTT colorimetric assay, and the results are presented in Figure 2. As illustrated in Figure 2 *S. nepeta* EO significantly reduced cell viability in a concentration-dependent manner. The EO exhibited a 50% lethal concentration (LC_50_) of 14.060 ± 1.364 µg mL^−1^. In comparison, the reference chemotherapeutic drug melphalan displayed a markedly lower LC_50_ of 2.129 ± 0.126 µg mL^−1^ (*p* < 0.0001), confirming its higher potency.

## 4. Discussion

The evaluation of *S. nepeta*’s biological properties stems from growing interest in plant-derived EOs as safer and more effective alternatives to synthetic agents. Despite its traditional use in folk medicine, the mechanistic basis of its therapeutic potential remains poorly understood. Therefore, exploring its antimicrobial and antiproliferative activities provides valuable insight into the pharmacological relevance of this species and its bioactive constituents.

First, we analyzed the chemical composition of *S. nepeta* EO and found that pulegone was the predominant compound. According to the literature, the yield and chemical composition of *Satureja nepeta* EO exhibits considerable quantitative and qualitative variability, largely influenced by environmental factors and differences in extraction procedures. Several studies have highlighted this variability across regions. For example, El Brahimi et al. reported an extraction yield of 2.80% for *S. nepeta* collected in the Taza province. In contrast, the low yield obtained in our study 0.076% (*w*/*w*) can be attributed to specific conditions affecting our plant material. The samples were collected outside the flowering period, when EO content is naturally reduced, and during a year of severe drought, a climatic stress known to suppress volatile metabolite production. Additionally, differences in drying conditions between studies may contribute to further losses of volatile constituents, thereby influencing the final yield [10].

Antioxidant assays are used to evaluate the ability of natural compounds to neutralize ROS and mitigate oxidative stress. These assays also play a crucial role in the development of safer, healthier products, such as functional foods, dietary supplements, and natural alternatives to synthetic antioxidants. By emphasizing the protective role and significance of antioxidants, these tests are essential for assessing the antioxidant activity of natural compounds and their potential in preventing diseases linked to oxidative stress [22,23,24]. During this study, we evaluated the antioxidant potential of *S. nepeta* EO, which demonstrated an immediate antioxidant response in its pure form, efficiently neutralizing DPPH and ABTS+ radicals and reducing both ferric and molybdenum complexes. Previous studies have reported significant quantitative variations in the antioxidant activity of *S. nepeta* EO. Brahimi et al. investigated the antioxidant activity of *S. nepeta* EO and reported an IC_50_ value of 23.03 ± 4.31 µg/mL for the DPPH assay and an EC_50_ value of 55.38 ± 2.26 µg/mL for the FRAP assay [6]. Similarly, Khodja et al. examined the antioxidant properties of *C. nepeta* from Algeria and reported an IC_50_ value of 8.33 ± 0.37 mg/mL for the DPPH assay, while the EC_50_ value for the FRAP assay was 10.1 mg/mL [9,25]. Formisano et al. evaluated the antioxidant activity of *S. nepeta* EO using DPPH and FRAP assays [11]. They obtained an IC_50_ and EC_50_ of 0.05 ± 0.01 mmol TE/L and 0.87 ± 0.06 mmol TE/L.

The antioxidant results may appear inconsistent at first glance, but this can be clarified by considering the distinct mechanisms underlying each assay. TAC measures the total reducing power of the oil under acidic conditions, capturing the overall electron-donating capacity of its constituents, whereas DPPH and ABTS evaluate radical-scavenging activity, which depends specifically on hydrogen-donating molecules. As a result, *S. nepeta* EO may display strong reducing capacity (high TAC) while showing lower radical-scavenging activity (low DPPH/ABTS). These observations also underscore how methodological variations such as solvent choice, extraction parameters, and assay protocols can significantly influence the antioxidant capacity reported for *S. nepeta* EO across different studies [26].

The agar diffusion assay revealed that *S. nepeta* EO possesses broad-spectrum antibacterial activity against both Gram-positive and Gram-negative bacteria. The inhibition zone diameters ranged from 14 mm to 27 mm, indicating variable strain sensitivity. The highest inhibition was observed for *Pseudomonas aeruginosa*, a Gram-negative bacterium typically characterized by high intrinsic resistance to antibiotics, followed by *Salmonella enterica* and *Staphylococcus aureus*. These results highlight the capacity of *S. nepeta* EO to act against clinically relevant pathogens associated with human and foodborne infections.

In recent decades, the increasing resistance of bacteria to antibiotics has posed a major threat to human and animal health, necessitating an urgent search for alternative therapeutic strategies [25]. Consequently, the development of novel treatments for bacterial infections has emerged as a promising and efficient approach, emphasizing the crucial role of natural sources in the discovery of antibacterial compounds. In this study, we evaluated the antibacterial activity of *S. nepeta* EO. The EO demonstrated broad-spectrum antibacterial activity, making it a valuable candidate for combating polymicrobial infections. Given the increasing prevalence of antibiotic resistance, *S. nepeta* EO could serve as a complementary or alternative therapeutic option, contributing to the development of novel antimicrobial strategies. We suggest that the EO constituents can effectively penetrate bacterial membranes or interfere with cellular metabolism. Such activity is commonly attributed to the high content of monoterpenes such as pulegone, menthol, and cis-pulegol, which are known to disrupt membrane integrity and induce leakage of intracellular contents. Although comparisons with standard antibiotics provide useful insights into the relative antibacterial performance of the EO, differences in physicochemical properties and diffusion behavior in agar media should be considered when interpreting inhibition zone diameters.

Comparative analysis with published data revealed both convergences and discrepancies. The low MIC obtained for *P. aeruginosa* aligns with the findings of Radi et al. [7] and Bouzidi et al. [10], though it is lower than those reported by Hayani et al. [8], indicating potential differences in the phytochemical composition or testing methodology. In contrast, the high MIC for *E. coli* suggests lower permeability or adaptive resistance mechanisms specific to this species. Among Gram-positive bacteria, the EO demonstrated substantial inhibitory activity against *S. aureus* and *B. subtilis*, consistent with previous reports of strong sensitivity of these strains to *Satureja* species oils. Notably, this study provides the first evidence of activity against *S. epidermidis* and *M. luteus*, both of clinical importance in biofilm formation and nosocomial infections. It suggests effective growth inhibition within a biologically relevant concentration range. Collectively, the results indicate that *S. nepeta* EO exerts a balanced antibacterial spectrum, with potent effects on both Gram-positive and Gram-negative strains. Such outcomes reinforce the potential of this oil as a natural antimicrobial resource and warrant further investigation into its mechanism of action and synergistic use against multidrug-resistant pathogens.

DNA gyrase B is an essential bacterial enzyme involved in ATP-dependent DNA supercoiling and replication, and is therefore considered a relevant target in antibacterial drug discovery [27]. In this context, an in silico docking analysis was performed to explore the potential interactions between the major compounds of *S. nepeta* and DNA gyrase B. The results suggested that all tested compounds were able to interact with the enzyme, although with varying predicted binding affinities. Among them, menthol exhibited the most favorable binding energies and the lowest estimated Ki values, indicating a comparatively higher predicted affinity for the target. Pulegone and cis-pulegol displayed slightly weaker binding energies but were also predicted to occupy the binding site of the enzyme. In addition, cis-pulegol formed hydrogen-bond interactions with the Gram-positive gyrase B model, whereas menthol showed similar predicted affinities toward both Gram-positive and Gram-negative targets.

Overall, the observed binding energies (approximately −5 to −6 kcal/mol) suggest moderate interactions between the tested compounds and DNA gyrase B. Although these findings do not demonstrate enzyme inhibition, they may indicate that gyrase B could represent one of several potential molecular targets contributing to the antibacterial activity of *S. nepeta* EO. The consistency between the docking predictions and the in vitro antibacterial activity observed against both Gram-positive and Gram-negative bacteria provides preliminary support for this hypothesis. Nevertheless, additional experimental studies, including enzyme inhibition assays, would be required to confirm the involvement of DNA gyrase B in the antimicrobial effects of the oil.

The antiproliferative effect observed in P3 cells corroborates previous evidence highlighting the antitumor potential of monoterpene-rich EOs. Monoterpenes such as pulegone, menthol, and cis-pulegol are reported to induce apoptotic and antiproliferative responses through mechanisms involving mitochondrial dysfunction, reactive oxygen species (ROS) generation, and disruption of calcium homeostasis [28]. Their lipophilic properties allow for facile penetration of cellular membranes, facilitating interactions with critical intracellular targets. Comparable studies support these findings. *Origanum vulgare* EO, containing 77.45% pulegone, displayed pronounced cytotoxicity against MCF-7, HeLa, Jurkat, HT-29, and T24 cell lines [29]. Menthol was shown to suppress tumor angiogenesis via TRPM8 activation, thereby preventing VEGF-induced TRPV1 trans-activation [30,31]. Similarly, *Satureja thymbra* EO, rich in α-pinene, p-cymene, thymol, and sabinene, demonstrated moderate cytotoxicity (IC_50_ = 154.3 ± 1.2 µg mL^−1^ for C32; 155.9 ± 1.1 µg mL^−1^ for ACHN) [32].

Taken together, these data suggest that *S. nepeta* EO contains bioactive monoterpenes with antiproliferative activity against the tested cancer cell line. However, it is important to emphasize that this effect was evaluated only on a single cancer cell line, without assessment on normal cells. Therefore, the selectivity of the cytotoxic effect remains to be established, and the selectivity index could not be determined in the present study. Further investigations are required to evaluate the differential effects of *S. nepeta* EO on malignant versus non-malignant cells, to clarify the underlying apoptotic mechanisms, and to confirm its efficacy in in vivo models. These additional studies will be essential to properly assess its therapeutic potential and safety before considering any application in functional food or pharmaceutical development.

## 5. Conclusions

This study underscores the potential of *S. nepeta* EO from the Al Haouz region of Morocco as a natural source of antioxidant, antimicrobial, and antiproliferative activities, driven primarily by pulegone and menthol. It should be noted, however, that oil’s yield and composition vary substantially with geographic origin, environmental conditions, phenological stage, and processing methods, meaning the reported activities are specific to this population and sampling context. Broader validation across multiple populations, alongside acute toxicity and healthy-cell selectivity studies, is still needed to confirm the safety profile and therapeutic promise of this EO in medical and biotechnological applications.

## Figures and Tables

**Table 1:** Molecular docking target of gyrase B, PDB IDs, and active site coordinates.

Target		PDB ID	Coordinates		
			X	Y	Z
Gyrase B	G^−^	1KZN	19.150	30.393	34.745
	G^+^	4URN	−31.684	8.021	−4.598

**Table 2:** Chemical composition of *S. nepeta* essential oil.

Components	Retention Time	Kovats Index		Percentage (%)	Molecular Formula
		KI Exp ^a^	KI Lit ^b^		
Menthol	10.525	1169	1167	6.64	C10H20O
Isopulegone	10.93	1180	1175	1.58	C10H16O
Neoisomenthol	11.22	1188	1179	0.57	C10H20O
cis-Pulegol	12.497	1223	1226	2.04	C10H18O
Pulegone	13.374	1243	1233	68.63	C10H16O
Piperitone	13.951	1257	1249	1.69	C10H16O
Isopiperitone	14.662	1275	1262	0.49	C10H14O
Spathulenol	26.707	1581	1577	1.71	C15H24O
Caryophyllene oxide	26.883	1586	1582	0.63	C15H24O
Total				83.98	

^a^ Linear retention index on BP-5 column, experimentally determined by using homologous series of C9-C20 alkanes. ^b^ Relative linear retention index literature taken from Adams for BP-5 capillary column.

**Table 3:** Antioxidant activities of *S. nepeta* essential oil.

	DPPH IC_50_ (mg/mL)	ABTS IC_50_ (mg/mL)	TAC EC_50_ (µg/mL)	FRAP EC_50_ (µg/mL)
Essential oil	10 ^b^≥	10 ^b^≥	3.747 ± 0.577 ^b^	10 ^b^≥
Ascorbic acid	3.409 ± 0.196 ^a^	3.679 ± 0.763 ^a^	7.42 ± 0.137 ^a^	8.127 ± 0.467 ^a^

Results are expressed as mean ± SD. Different letters indicate a significant difference between essential oil and ascorbic acid for each test, as determined by Student’s *t*-test (*p* < 0.05).

**Table 4:** Determination of diameter of the inhibition zone (DIZ) and minimum inhibitory concentration (MIC) of *S. nepeta* essential oil.

Bacterial Strains	*S. nepeta* Essential Oil			Chlortetracycline	Cefotaxime	Chloramphenicol
	DIZ (mm)	MIC (µg/mL)	Activity Index	DIZ		
*E. coli*	14 ± 0 ^b^	104	0.77	18 ± 0 ^a^	-	-
*S. aureus*	21 ± 0 ^b^	13.104	0.77	27 ± 0 ^a^	-	-
*S. epidermis*	20 ± 0 ^b^	13.10	0.76	26 ± 0 ^a^	-	-
*M. luteus*	17 ± 0 ^b^	26	0.77	25 ± 0 ^a^	-	-
*P. aeruginosa*	27 ± 2.64 ^b^	2.375	0.87	31 ± 1 ^a^	-	-
*S. enterica*	21.16 ± 0.28 ^b^	3.25	0.84	-	25 ± 0 ^a^	-
*B. subtilus*	15.1 ± 0.17 ^b^	13	0.83	-	-	18 ± 0 ^a^

Different letters indicate statistically significant differences compared to the control group, as determined by Student’s *t*-test (*p* < 0.0001). The antibacterial activity of *S. nepeta* EO was evaluated against chlortetracycline and cefotaxime in *E. coli*, *S. aureus*, *S. epidermidis*, *M. luteus*, *P. aeruginosa*, and *S. enterica*, respectively, and against chloramphenicol in *B. subtilis*. The DIZ values include the diameter of the disc (6 mm).

**Table 5:** Docking results of the major compounds of *S. nepeta* essential oil on gyrase B. Binding free energies (ΔGb, kcal/mol), inhibition constants (Ki, µM), Root Mean Square Deviation (RMSD), and hydrogen-bond interactions are reported for Gram-positive (G^+^) and Gram-negative (G^−^) bacterial targets.

Compounds	Gyrase B					
	G^+^			G^−^		
	ΔGb (Kcal/mol)	Ki (µM)	H-Bond	ΔGb (Kcal/mol)	Ki (µM)	H-Bond
Cis-pulegol	−5.11	179.09	GLU A:53, ASN A:56	−5.28	135.89	
Menthol	−6.09	34.41	ASN A:49	−5.34	121.67	ASN A:46
Pulegone	−5.62	75.87	-	−5.35	119.88	

## Data Availability

All data supporting the findings of this study are contained within the article.

## Abbreviations

The following abbreviations are used in this manuscript:

AA	Ascorbic acid
ABTS	2,2-azino-bis(3-ethylbenzothiazoline-6-sulfonic acid)
ADT	AutoDock Tools
AI	Activity index
ANOVA	Analysis of variance
*B. subtilis*	*Bacillus subtilis*
CFU	Colony-forming unit
DIZ	Diameter of inhibition zone
DMSO	Dimethyl sulfoxide
DPPH	2,2-diphenyl-1-picrylhydrazyl
*E. coli*	*Escherichia coli*
EC_50_	Half-maximal effective concentration
EO	Essential oil
FCS	Fetal calf serum
FRAP	Ferric-reducing antioxidant power
GC-MS	Gas chromatography–mass spectrometry
IC_50_	Half-maximal inhibitory concentration
Ki	Inhibition constant
LC_50_	Half-maximal lethal concentration
*M. luteus*	*Micrococcus luteus*
MH	Mueller–Hinton
MIC	Minimal inhibitory concentration
MTT	3-(4,5-dimethylthiazol-2-yl)-2,5-diphenyltetrazolium bromide
*P. aeruginosa*	*Pseudomonas aeruginosa*
PDB	Protein Data Bank
ROS	Reactive oxygen species
RPMI	Roswell Park Memorial Institute
*S. aureus*	*Staphylococcus aureus*
*S. enterica*	*Salmonella enterica*
*S. epidermidis*	*Staphylococcus epidermidis*
*S. nepeta*	*Satureja nepeta*
TAC	Total antioxidant capacity
ΔGb	Binding free energy
